# The Role of Artificial Intelligence and Emerging Technologies in Advancing Total Hip Arthroplasty

**DOI:** 10.3390/jpm15010021

**Published:** 2025-01-09

**Authors:** Luca Andriollo, Aurelio Picchi, Giulio Iademarco, Andrea Fidanza, Loris Perticarini, Stefano Marco Paolo Rossi, Giandomenico Logroscino, Francesco Benazzo

**Affiliations:** 1 Sezione di Chirurgia Protesica ad Indirizzo Robotico—Unità di Traumatologia dello Sport, Ortopedia e Traumatologia, Fondazione Poliambulanza, 25124 Brescia, Italy; 2 Ortopedia e Traumatologia, Università Cattolica del Sacro Cuore, 00168 Rome, Italy; 3 Artificial Intelligence Center, Alma Mater Europaea University, 1010 Vienna, Austria; 4 Unit of Orthopedics, Department of Life, Health and Environmental Sciences, University of L’Aquila, 67100 L’Aquila, Italy; 5 Department of Life Science, Health, and Health Professions, Università degli Studi Link, Link Campus University, 00165 Rome, Italy; 6 Biomedical Sciences Area, IUSS University School for Advanced Studies, 27100 Pavia, Italy

**Keywords:** total hip arthroplasty, hip surgery, artificial intelligence, THA, augmented reality, virtual reality

## Abstract

Total hip arthroplasty (THA) is a widely performed surgical procedure that has evolved significantly due to advancements in artificial intelligence (AI) and robotics. As demand for THA grows, reliable tools are essential to enhance diagnosis, preoperative planning, surgical precision, and postoperative rehabilitation. AI applications in orthopedic surgery offer innovative solutions, including automated hip osteoarthritis (OA) diagnosis, precise implant positioning, and personalized risk stratification, thereby improving patient outcomes. Deep learning models have transformed OA severity grading and implant identification by automating traditionally manual processes with high accuracy. Additionally, AI-powered systems optimize preoperative planning by predicting the hip joint center and identifying complications using multimodal data. Robotic-assisted THA enhances surgical precision with real-time feedback, reducing complications such as dislocations and leg length discrepancies while accelerating recovery. Despite these advancements, barriers such as cost, accessibility, and the steep learning curve for surgeons hinder widespread adoption. Postoperative rehabilitation benefits from technologies like virtual and augmented reality and telemedicine, which enhance patient engagement and adherence. However, limitations, particularly among elderly populations with lower adaptability to technology, underscore the need for user-friendly platforms. To ensure comprehensiveness, a structured literature search was conducted using PubMed, Scopus, and Web of Science. Keywords included “artificial intelligence”, “machine learning”, “robotics”, and “total hip arthroplasty”. Inclusion criteria emphasized peer-reviewed studies published in English within the last decade focusing on technological advancements and clinical outcomes. This review evaluates AI and robotics’ role in THA, highlighting opportunities and challenges and emphasizing further research and real-world validation to integrate these technologies into clinical practice effectively.

## 1. Introduction

Total hip arthroplasty (THA) is a widely performed surgical procedure that has undergone substantial growth and innovation. Currently, approximately 7 million hip and knee replacements have been performed in the United States, with the number of arthroplasties increasing annually. This upward trend is expected to continue in the coming years [1].

The digital age has brought an increasing demand for reliable and accessible medical information. Artificial intelligence (AI) has emerged as a transformative tool in addressing this need, offering innovative approaches to support patients, caregivers, and healthcare professionals. AI-powered systems, including advanced language models, are being developed to provide accurate, evidence-based information on medical procedures such as THA. These technologies have the potential to enhance patient education, guide clinical decision-making, and support research in orthopedic surgery [2,3].

Patients considering THA require comprehensive information regarding the surgical technique, expected outcomes, associated risks, and postoperative recovery. Similarly, healthcare providers rely on precise, up-to-date data to inform patient care and optimize surgical planning. AI applications in orthopedics extend beyond education to include clinical tools that assist surgeons in decision-making, particularly in the context of robotic-assisted surgery, which represents a significant advancement in joint replacement [4,5].

Robotic-assisted hip arthroplasty has revolutionized surgical practice by integrating advanced preoperative planning tools, intraoperative precision, and real-time feedback mechanisms. This approach enhances surgical accuracy, minimizes complications, and accelerates recovery. Indications for robotic-assisted procedures include complex anatomical cases, younger and more active patient populations, and revision surgeries, where enhanced precision is especially beneficial [6].

However, the adoption of robotic systems in orthopedic surgery is not without challenges. Financial constraints, a steep learning curve for surgeons, and limitations in certain clinical scenarios must be addressed. Additionally, the cost-effectiveness and scalability of these technologies remain subjects of ongoing investigation.

Beyond robotics, AI plays a growing role in medical education and research, offering tools for synthesizing large volumes of data and supporting evidence-based practice. Despite its promise, AI systems face limitations, including reliance on the quality and timeliness of training data, which may lead to outdated or inaccurate information. Furthermore, AI lacks the contextual insight and personalized learning experience provided by experienced educators, which can result in oversimplification or misinterpretation of complex concepts [7,8].

This study seeks to explore the potential role of AI in THA, with a focus on its ability to support traditional medical knowledge and contribute to advancements in surgical techniques and rehabilitation. By assessing AI-generated data’s reliability and relevance in patient care and clinician support, this research aims to outline the opportunities and limitations of AI in conveying medical information. The findings may help improve the understanding of how AI can complement existing approaches to healthcare delivery in the context of evolving technology.

To ensure a comprehensive understanding of the role of AI and emerging technologies in advancing THA, a structured literature search was conducted. The primary databases accessed included PubMed, Scopus, and Web of Science. Keywords such as “artificial intelligence”, “machine learning”, “robotics”, “emerging technologies”, and “total hip arthroplasty” were used in various combinations. Inclusion criteria were set to focus on peer-reviewed articles published in English within the last 10 years, emphasizing studies reporting technological advancements, clinical outcomes, and future trends in THA. Exclusion criteria included non-peer-reviewed articles, conference abstracts, and studies not directly related to THA. This strategy aimed to capture the most relevant and up-to-date evidence for the narrative review, ensuring both depth and reliability.

Table 1 presents an overview introducing the use of artificial intelligence and emerging technologies in advancing total hip arthroplasty.

## 2. Hip Osteoarthritis Diagnosis, Preoperative Planning, and Risk Stratification

AI demonstrated potential in advancing the diagnosis and preoperative planning for orthopedic conditions, particularly in hip osteoarthritis (OA) and THA [39]. The application of AI to medical imaging, including radiographs (X-rays), computed tomography (CT), and magnetic resonance imaging (MRI), has led to a revolution in automating and refining complex decision-making processes, improving both diagnostic accuracy and surgical planning [40,41]. One of the key areas where AI is making an impact is in the automated grading of hip OA severity [9]. Traditionally, international classifications have been subjective, relying on manual assessments. However, deep learning models, like those developed by Masuda et al., are capable of automatically grading OA severity based on digitally reconstructed radiographs (DRRs) from CT scans [9]. These AI models not only predict disease severity but also incorporate uncertainty estimation, providing an additional layer of reliability in the diagnostic process and helping clinicians assess the potential for classification errors, particularly in complex cases [10].

In line with these advancements, studies such as the Hip OA-Grading project have demonstrated the capability of deep learning models to analyze and grade hip OA severity with high accuracy. These models integrate regression and classification methods, achieving significant performance metrics such as an exact class accuracy of approximately 0.65 and a one-neighbor class accuracy of 0.95. Additionally, the incorporation of model uncertainty ensures that predictions are robust, aiding clinicians in identifying cases where further manual review or advanced imaging may be warranted. This dual approach of accurate grading and uncertainty management represents a critical advancement in diagnostic workflows for hip OA [11].

In the realm of preoperative planning, AI models are increasingly used to assist surgeons in making informed decisions regarding implant placement and the management of patients undergoing THA [12]. Jang et al. demonstrated the potential of AI to predict the hip joint center (HJC) from anteroposterior pelvis radiographs, achieving rapid and accurate results that can significantly aid in implant positioning [42]. The integration of such tools into clinical workflows can reduce the subjectivity and variability associated with traditional planning methods, ensuring more consistent outcomes.

Moreover, AI’s role extends beyond diagnosis and planning to risk stratification. Predicting postoperative complications is crucial to improving patient outcomes, and recent advancements have shown how AI can be leveraged for this purpose. Shah et al. developed a machine learning algorithm that predicts the likelihood of major complications after THA, outperforming traditional models like logistic regression [22]. By incorporating various preoperative factors, such as comorbidities and demographic information, the AI model offers a personalized risk assessment, which enhances decision-making and facilitates more tailored patient care [13].

AI’s capacity to handle vast amounts of data, such as radiographs from multiple views or even 3D scans, makes it an invaluable tool for orthopedic surgeons. Models can now be trained to analyze CT scans, MRI, and X-rays, providing comprehensive evaluations of hip joint anatomy, implant compatibility, and disease progression. The ability of AI to process various imaging modalities and views significantly improves the precision and efficiency of preoperative planning [14].

AI-based tools also introduce the ability to quantify uncertainty in predictions, which is crucial in instrumental settings where decision-making is based on high-stakes outcomes [43]. By detecting potential outliers and flagging uncertain predictions, AI systems help surgeons make informed decisions, particularly when dealing with complex or rare cases [10].

AI technology continues to evolve, and the integration of tools into clinical practice promises to streamline processes, reduce human error, and improve patient outcomes. The ability to automate complex tasks such as implant design identification, risk assessment, and OA grading allows surgeons to focus more on clinical decision-making rather than manual analysis. Moreover, the development of hybrid models that combine various machine learning approaches will further increase the accuracy, speed, and reliability of AI in orthopedic care [44].

The advancement of AI in orthopedics will likely lead to more personalized and efficient care, from diagnosis to postoperative management. AI has also been instrumental in enhancing the preoperative planning process for THA. As part of this, deep learning models have been developed to automate the identification and segmentation of the hip joint, which is essential for accurate implant positioning and simulation. For example, Wu et al. introduced Changmugu Net (CMG Net), a deep learning network that significantly outperformed traditional methods for femur and pelvis segmentation from CT images [15]. Their model achieved high accuracy in terms of the Dice coefficient (93.55%) and reduced segmentation time to just under 26 s, which is crucial for streamlining preoperative workflows. This technological advancement demonstrates AI’s ability to improve both the accuracy and efficiency of preoperative planning, especially in cases involving complex or severely diseased joints.

Additionally, hybrid models that integrate AI-based implant design identification and automated OA severity grading systems hold promise for advancing holistic preoperative assessments. These tools can synergize implant selection processes with disease grading, offering surgeons a unified platform for comprehensive planning. For instance, incorporating digitally reconstructed radiographs and uncertainty estimation into such workflows could further improve surgical precision while minimizing potential errors [45].

AI’s ability to predict postoperative complications also holds promise in improving THA outcomes. The work of Bulloni et al. focused on developing AI models that predict hip implant failure using radiological data from X-rays and CT scans, analyzing the evolution of periprosthetic bone and implant positioning over time [46]. Their hybrid models, which incorporate both traditional and evolutionary radiological features, demonstrated an area under the ROC curve (AUC) of 0.94, making them highly effective in predicting complications. This ability to forecast complications in advance allows for more proactive patient management, potentially reducing the incidence of postoperative issues.

These AI models provide clinicians with accurate, reproducible grading that can help in tailoring individualized treatment plans, from conservative management to surgical interventions like THA.

The integration of AI in medical imaging, such as CT, MRI, and radiographs, is transforming the preoperative landscape. AI’s capability to automatically segment and analyze complex anatomical structures not only reduces the time spent on manual assessments but also increases the precision of these evaluations. Furthermore, the introduction of uncertainty estimation, as demonstrated in several studies, allows AI to provide an indication of how confident it is in its predictions [23]. This can be particularly helpful in clinical settings where high-stakes decisions are being made, such as when planning complex surgeries or managing high-risk patients.

The future of AI in orthopedic care lies in further refinement of these models, the incorporation of multimodal data (such as combining imaging data with clinical and genetic information), and continued real-world validation. As these models continue to improve, AI will likely become an indispensable tool in orthopedic practice, not only in diagnosis and preoperative planning but also in guiding postoperative care, optimizing implant choices, and predicting long-term outcomes. Ultimately, the ongoing evolution of AI technologies promises to make orthopedic care more personalized, efficient, and precise.

## 3. Implant Identification

AI has shown significant promise in enhancing the accuracy and efficiency of implant identification in THA, particularly in the context of preoperative planning and revision surgeries. The identification of implant designs from radiographs is a crucial task for surgeons, especially when managing complex cases that involve the replacement or retention of failed implants [24]. Traditionally, this process relied on manual recognition of femoral and acetabular components, a task that is not only time-consuming but also prone to human error. Recent advancements in deep learning, specifically convolutional neural networks (CNNs), have revolutionized the field by automating implant recognition, providing an invaluable tool for assisting orthopedic surgeons in both primary and revision arthroplasties [25].

Several studies have demonstrated the remarkable performance of AI in identifying a wide range of implant designs from plain radiographs. In a study by Karnuta et al., a deep learning model was able to classify femoral-sided THA implants from a dataset of nearly 3000 anteroposterior radiographs [26]. The model achieved a mean accuracy of 97.9% in external testing, with high sensitivity and specificity, marking a significant step toward the clinical application of AI in implant identification. Similarly, the work of Borjali et al. showed that CNN models could achieve 100% accuracy in identifying common implant designs, outperforming manual methods and significantly reducing identification time to just seconds per image [47].

Beyond accuracy, the use of AI tools like Total Hip Arthroplasty Automated Implant Detector (THA-AID) introduces the ability to detect uncertainty in predictions and flag outlier data, enhancing the reliability of AI-based decisions in clinical practice [27]. This level of confidence in AI predictions is especially important when dealing with rare or complex implant designs, as the AI system can signal to the surgeon when the identification is uncertain, prompting further investigation. This approach not only ensures that clinicians make informed decisions but also helps avoid potential complications during surgery [48].

Moreover, the ability of AI systems to process and identify implants across multiple radiographic views (e.g., lateral and oblique) further increases their utility. In contrast, traditional manual methods typically rely on limited image perspectives, which can sometimes obscure key features of the implant. AI models can analyze various angles, allowing for more accurate and comprehensive implant identification. Studies such as those by Borjali et al. and Klemt et al. have showcased the application of deep learning models to identify both femoral and acetabular components from various radiographic views, significantly enhancing the model’s robustness [28,49].

The integration of AI into clinical workflows offers several advantages over traditional methods. For one, AI systems can drastically reduce the time required to identify an implant, which is crucial in time-sensitive clinical environments [50]. In cases of failed THA, where accurate implant identification is essential for revision surgery planning, the ability to make quick decisions can improve patient outcomes by reducing surgical delays and the likelihood of errors. AI systems can also provide an objective and standardized method of implant classification, reducing variability that can arise from human interpretation [51].

Furthermore, AI’s role in assisting preoperative planning goes beyond just identification. By accurately identifying the implant design, AI can assist surgeons in selecting the most appropriate replacement components, particularly in complex revision surgeries where retaining well-fixed components is essential [29]. The rapid identification of implant designs can also streamline communication between surgeons and other healthcare providers, including those involved in implant procurement and inventory management.

While AI has demonstrated significant promise in this domain, the continued development of these technologies is crucial. The ongoing expansion of implant libraries, the incorporation of more diverse datasets, and the integration of multimodal image processing (e.g., CT or MRI scans in addition to radiographs) will only enhance the accuracy and applicability of AI models [52]. Additionally, the inclusion of AI tools in orthopedic education and clinical practice requires rigorous validation and real-world testing to ensure their safe and effective use [51].

## 4. The Impact of Robotics on Surgical Precision: Innovations and Outcomes

In recent years, robotic and computer-assisted technologies have significantly transformed the field of THA, offering new avenues to optimize surgical precision and patient outcomes. These innovations, including robotic systems like MAKO and TSolution-One, and computer-assisted navigation, aim to address the limitations of manual THA (mTHA) by improving implant positioning, reducing complications, and enhancing long-term clinical results. Through advanced imaging techniques such as CT scanning and 3D reconstruction, these systems enable individualized preoperative planning and intraoperative feedback, which help surgeons achieve higher accuracy in implant placement and overall surgical reproducibility [16,17].

A key advantage of these technologies lies in their ability to restore hip mechanics with greater precision, reducing risks such as dislocation, instability, and edge loading. By providing real-time quantitative data on leg length, offset, and impingement modeling, robotic and navigated systems ensure reproducible outcomes, even in complex cases involving high-risk patients, abnormal spinopelvic mobility, or severe developmental dysplasia of the hip [53,54]. Research has consistently demonstrated that robotic THA (rTHA) outperforms manual techniques in maintaining implants within safe inclination and anteversion zones, achieving near-perfect accuracy in certain studies [18,55].

The benefits of robotic-assisted THA are particularly notable in reducing complication rates. Dislocation, historically occurring in 1–4% of cases, is significantly reduced in robotic procedures, with hazard ratios for revision rates ranging between 0.3 and 0.46 in various populations [56,57]. Additionally, rTHA minimizes leg length discrepancies, a common source of postoperative dissatisfaction, and enhances bone stock preservation by enabling controlled and precise bone removal [58]. Clinical outcomes, such as patient-reported satisfaction and functional recovery, also show promising trends, with robotic procedures yielding higher scores on metrics like the Harris Hip Score (HHS) and the Forgotten Joint Score (FJS) [19].

Despite these advantages, the integration of robotic and navigated systems in THA has presented several challenges. Increased operative times, largely attributed to the surgeon’s learning curve, remain a concern, with robotic cases taking an average of 10–20 min longer than manual procedures [59]. However, studies indicate that proficiency is achieved after 12–35 cases, after which operative times become comparable to conventional methods [21,60]. Another limitation is cost; robotic systems require substantial upfront investment, increased hospital charges, and ongoing maintenance expenses. For instance, preoperative CT scans, necessary for robotic planning, add to overall costs and expose patients to additional radiation [16,20]. Nevertheless, cost–benefit analyses suggest that the reduction in complications and revision surgeries offsets these expenses, with robotic-assisted THA potentially saving USD 945 for Medicare patients and USD 1810 for private payers over five years [20].

The adoption of robotic and navigated THA has seen a steady increase, albeit at a slower pace compared to robotic knee arthroplasty. Data from national registries indicate an upward trend, with the percentage of computer-navigated THA procedures rising from 0.1% to 1.9% in the U.S. between 2005 and 2018 and from 1.9% to 4.4% in Australia between 2009 and 2019 [61,62]. Public interest in robotic arthroplasty has also grown, as evidenced by increased online search trends [63]. However, widespread adoption is hindered by financial and logistical barriers, as well as the need for specialized training [60,64].

While the short-term functional benefits of robotic systems remain less clear, radiological outcomes demonstrate significant advantages. For instance, the MAKO system has shown superior precision in achieving optimal cup anteversion and inclination, with significantly higher percentages of components within safe zones compared to manual techniques [17,65]. This enhanced accuracy is particularly valuable in complex revision surgeries, where distorted bony anatomy or absent landmarks complicate alignment. Studies have reported a 0% dislocation rate in a series of 72 revision THAs performed using computer navigation [66]. However, discrepancies between preoperative planning and postoperative outcomes, such as deviations in cup inclination and anteversion, highlight areas for further improvement [17].

Robotic systems also contribute to long-term outcomes by addressing wear-related complications. Advances in bearing surfaces, such as highly cross-linked polyethylene (HXLPE), complement the precision of robotic surgery by reducing osteolysis and enabling the use of larger femoral heads, which further decrease dislocation risks [67,68]. These technological advancements, combined with minimally invasive approaches, represent a holistic effort to optimize patient care, particularly for younger, more active populations who demand durable outcomes.

The potential of robotic THA to improve both clinical and economic outcomes is substantial, but its success hinges on continued research, refinement of technology, and broader accessibility. While radiological precision and reduced complication rates are clear benefits, long-term studies are necessary to determine whether these advantages translate into meaningful improvements in functionality and quality of life [17]. Factors such as surgical volume, patient characteristics, and institutional infrastructure must also be considered to assess the broader applicability of these systems [37,69].

Robotic and navigated THA technologies have demonstrated measurable benefits in improving surgical precision, reducing complication rates, and enhancing patient outcomes [70]. Despite challenges such as cost, learning curves, and logistical barriers, these innovations represent a pivotal shift in orthopedic surgery. As these systems continue to evolve, their role in ensuring reproducible and durable outcomes will likely become even more integral, paving the way for the next generation of advanced surgical care.

## 5. Virtual and Augmented Reality

The use of virtual reality (VR) and augmented reality (AR) represents a promising technological advancement in orthopedic surgery, particularly for hip-related procedures. These technologies, which rely on fully immersive virtual environments and overlaid real-world content, offer significant advantages in preoperative simulation, intraoperative navigation, and postoperative rehabilitation. This systematic review aims to examine the state of the art regarding the application of VR and AR in hip surgery and to summarize their benefits, limitations, and potential future applications [71,72].

One of the main findings is that VR and AR significantly enhance surgical training and procedural planning. VR, for instance, allows surgeons to perform repetitive skill-based training in a risk-free environment, which is particularly beneficial for mastering complex procedures like THA. Studies have demonstrated that VR-based training not only improves surgical precision but also accelerates the learning curve for trainees. For example, simulation programs like the HipNav system allow surgeons to plan and visualize acetabular cup placement with high accuracy, minimizing the risks of complications such as impingement or component malposition [73].

AR technology further enhances intraoperative accuracy by superimposing virtual anatomical models onto the surgical field, providing real-time guidance. Devices like the AR-HIP system have been shown to improve the precision of acetabular cup placement during THA, ensuring better alignment and reducing the likelihood of revision surgeries [74]. Additionally, AR-based navigation systems, such as those using HoloLens headsets, allow surgeons to track instruments and implants in real time, offering invaluable feedback during procedures [75]. This advantage is further supported by a systematic review and meta-analysis comparing AR-assisted THA with conventional methods. The review analyzed five studies involving 396 patients (200 AR-assisted and 196 conventional THA) and found that AR-assisted THA significantly improved the accuracy of acetabular cup inclination (SMD = −0.51, 95% CI [−0.96 to −0.07], *p* = 0.02) and anteversion (SMD = −0.96, 95% CI [−1.19 to −0.72], *p* < 0.00001) without increasing surgical time or intraoperative blood loss [76]. These findings underline AR’s potential to improve procedural precision while maintaining surgical efficiency.

In trauma surgery, VR and AR have demonstrated their utility in managing pelvic fractures and hip fractures. For example, VR simulations have been used effectively for training surgeons in percutaneous iliosacral screw placement, significantly reducing intraoperative radiation exposure and improving the accuracy of screw placement [77]. Similarly, AR-guided navigation systems have been tested for sacroiliac screw insertion, showing promising results in terms of accuracy and reduced operative times [78]. Furthermore, AR offers practical advantages over traditional navigation methods, such as fluoroscopy and surgical robots, by minimizing X-ray exposure, reducing equipment needs, and integrating seamlessly into the surgical workflow [79].

Beyond surgical training and intraoperative applications, these technologies have shown potential in postoperative rehabilitation. VR-based home rehabilitation systems provide patients with engaging, interactive exercises that can be performed remotely, addressing barriers such as limited access to physiotherapy services. Studies have shown that VR rehabilitation programs produce comparable functional outcomes to traditional methods, with the added advantage of convenience and cost-effectiveness [30]. Moreover, AR applications are being explored for guiding patient-specific rehabilitation programs, further enhancing recovery outcomes [80].

Despite these benefits, the implementation of VR and AR technologies in hip surgery is not without challenges. One significant barrier is the limited familiarity and acceptance of these technologies among surgeons, many of whom still prefer traditional learning methods. Furthermore, current simulators often lack the ability to replicate soft tissue manipulation, which is crucial for realistic surgical training. Cost and technological limitations also pose hurdles to widespread adoption [81]. The systematic review of AR-assisted THA also noted limitations, including small sample sizes, the inclusion of both randomized controlled trials (RCTs) and retrospective cohort studies, and significant heterogeneity among studies. Additional high-quality, long-term RCTs are needed to validate the benefits of AR-assisted THA on clinical outcomes and postoperative complications [82].

Looking forward, the development of multimodal simulators that combine tactile, visual, and auditory feedback could revolutionize surgical education. These tools would provide an immersive environment for mastering technical skills and translating them directly to the operating room. Additionally, as AR systems improve in their ability to account for organ deformation and dynamic interactions during surgery, their reliability and integration into routine clinical practice are likely to increase [83]. Mixed reality (MR) technologies, which merge elements of VR and AR, could further enhance intraoperative navigation and address some current limitations [59].

The role of these technologies in postoperative rehabilitation also holds great promise. Remote VR- and AR-based rehabilitation programs could address the growing demand for accessible, high-quality care, especially in the wake of challenges such as the COVID-19 pandemic. These programs not only reduce the need for travel but also allow clinicians to monitor and adapt rehabilitation plans in real time, ensuring optimal recovery outcomes [31].

VR and AR are poised to transform the landscape of hip surgery by enhancing surgical training, improving intraoperative precision, and supporting effective rehabilitation. While further comparative studies are needed to assess their cost-effectiveness and long-term clinical impact, the potential of these technologies to improve patient outcomes and streamline surgical education is undeniable. Future research should focus on integrating these tools into broader orthopedic practices and addressing current limitations to maximize their utility in clinical settings.

## 6. Postoperative Care and Rehabilitation

Orthopedic rehabilitation plays a critical role in restoring impaired function following trauma or surgery. Effective rehabilitation necessitates the strategic integration and progression of targeted exercises aimed at enhancing joint mobility and muscle strength, ultimately facilitating the recovery of physical function [84,85,86]. Contemporary rehabilitation strategies encompass both guided and independent exercise programs; however, technological advancements are revolutionizing this domain. Innovations such as VR, AR, gamification, and telerehabilitation are emerging as promising tools for enhancing the recovery process in orthopedic patients. Furthermore, the rapid evolution of electronic communication technologies, particularly in response to the challenges posed by the COVID-19 pandemic, has driven the accelerated adoption of the ‘Internet + Medical Treatment’ model, paving the way for novel approaches in rehabilitation and healthcare delivery [87].

This innovative method of healthcare delivery has partially mitigated challenges associated with accessing medical consultations and offers a novel framework for facilitating postoperative rehabilitation in orthopedic patients [88].

Recent RCTs have demonstrated improved adherence to home-based rehabilitation programs delivered through internet-based self-monitoring systems via mobile phones in patients with hemophilia-related knee dysfunction and other musculoskeletal conditions [89,90].

The utilization of a mobile application enhanced adherence to prescribed exercise regimens; however, it did not demonstrate significant improvements in physical performance, self-efficacy, or a reduction in caregiver burden when compared to a conventional home-based rehabilitation program for elderly patients recovering from hip fractures. Further research is warranted to explore the potential benefits of mobile applications in this context [32].

Telemedicine leverages network communication technology to establish a dynamic communication platform between orthopedic surgeons and their patients. This approach enables real-time remote feedback and guidance throughout the rehabilitation process, achieving outcomes that surpass the limitations of traditional follow-up methods and representing an innovative avenue for postoperative rehabilitation in orthopedic care [91].

Karlon et al. [33] evaluated telerehabilitation as a complementary modality to standard physical therapy and conducted a study involving patients undergoing hip surgery. The findings demonstrated that the telerehabilitation group exhibited consistent improvement across all outcome measures during follow-up, with particularly notable gains in the 2 min walking test (86.1%) and walking speed (65.6%). In contrast, no significant changes were observed in the control group during follow-up.

Most studies have focused on patients aged 40 to 60 years, with a majority possessing a university-level education. Age and social context significantly influence adaptability to technology; younger patients tend to exhibit greater predisposition and ease in utilizing such tools, whereas older patients often encounter challenges in engaging with technological platforms.

Emerging technologies hold significant potential to address the increasing demand for orthopedic rehabilitation. However, numerous challenges and limitations persist within the field.

The precision of these instruments is contingent upon the sophistication of the sensors and algorithms employed. In some cases, limitations in capturing the complete spectrum of motion or errors in activity interpretation may result in potential inaccuracies [92]. An additional challenge involves ensuring that patients consistently utilize these devices and perform their exercises accurately [93]. Research by Argent et al. addressing the issue of patient noncompliance revealed nonadherence rates ranging between 30% and 50% among individuals with musculoskeletal conditions [94]. A more recent systematic review evaluating strategies aimed at enhancing adherence across diverse musculoskeletal and medical populations reported an average compliance rate of 67% with prescribed home exercise regimens (based on 12 studies) [95]. A frequently referenced study by Sluijs et al. determined that adherence to physiotherapy among patients is suboptimal but refrained from making definitive conclusions regarding the extent of noncompliance, citing the absence of robust and reliable measurement tools [96]. Another issue arises from a lack of objective assessment data on postoperative gait, leading patients to maintain the compensatory walking patterns developed pre-operatively to mitigate pain associated with the arthritic joint. Consequently, this perpetuates gait abnormalities following surgery. To address this, patients must retrain their walking mechanics by strengthening weakened musculature. Persisting with improper gait patterns often results in continued joint strain and incorrect muscle utilization, potentially causing falls, reduced physical activity, or pain in other joints [97].

GS is a CE-marked Class 1 digital medical device that integrates patient-linked motion sensors with an artificial intelligence algorithm to generate objective gait data. This facilitates the delivery and monitoring of personalized rehabilitation programs with enhanced precision [34].

Elderly individuals constitute a large proportion of orthopedic patients, yet the adoption of VR, AR, gamification, and telerehabilitation remains limited among this demographic. Older patients often exhibit low receptivity to novel technologies and demonstrate limited adaptability. The primary challenge lies in enhancing these technologies to ensure they are accessible, user-friendly, and tailored to the needs of this population.

Patients’ compliance with treatment and clinical outcomes can be influenced by their perception of technology. Given that the majority of orthopedic patients are in the middle-to-older age range, it is essential to ensure the user-friendliness of remote virtual rehabilitation platforms. To address this need, increasingly simple and accessible platforms have been developed, eliminating the requirement for complex software or multidirectional camera setups. Patients only need access to a computer or smartphone with an internet connection to utilize these technologies effectively [98].

The integration of emerging technologies into orthopedic rehabilitation has the potential to significantly enhance outcomes for elderly patients. However, a critical challenge lies in accommodating individuals with limited adaptability to technological interfaces. As the aging population continues to grow, addressing this barrier is essential to ensure equitable access and efficacy of advanced rehabilitation solutions.

Elderly patients often face difficulties with complex technological systems due to age-related declines in cognitive and motor functions, as well as a lack of prior exposure to digital devices [99]. To address these challenges, emerging technologies must prioritize user-centered design. Simplified interfaces, intuitive controls, and clear instructional materials tailored to the needs of older adults are crucial. For instance, rehabilitation devices can incorporate large, easy-to-read displays, voice guidance, and minimalistic designs to reduce cognitive load and enhance usability. Automated feedback mechanisms, delivered through straightforward and accessible channels, can guide users in performing exercises correctly while minimizing the need for extensive caregiver intervention [35].

AI can also play a transformative role by offering personalized rehabilitation programs that adapt to the unique capabilities and limitations of elderly patients. For example, AI algorithms can analyze patient data to suggest modifications to exercise routines, ensuring safety and efficacy. However, it is imperative to present AI-driven insights in a manner that is easily comprehensible to both patients and their caregivers, fostering trust and adherence [100].

Another critical aspect is addressing the digital divide. Training programs and supportive resources are essential to empower elderly patients to engage with these technologies confidently. This may include in-person or virtual tutorials, assistance from healthcare professionals, and user-friendly manuals. Family members and caregivers can also be integrated into the rehabilitation process, acting as facilitators to bridge the gap between the patient and the technology [101].

Despite these advancements, cost and accessibility remain significant concerns. Many elderly individuals, particularly those in low-resource settings, may lack access to high-cost rehabilitation technologies. Policymakers and healthcare providers must work collaboratively to subsidize costs and ensure that these innovations are available to all socio-economic groups. Additionally, scaling down features to produce cost-effective versions of high-tech devices can broaden their reach without compromising functionality.

Remote orthopedic rehabilitation contributes to cost reduction within the national healthcare system. As demonstrated in other medical disciplines, these cost savings are primarily associated with decreased transportation needs, fewer hospital admissions, and lower rates of readmission [31,102].

The capacity to ambulate is fundamental for numerous daily activities, and an increasing proportion of elderly individuals undergo orthopedic interventions to regain mobility. Consequently, THA has emerged as one of the most frequently performed procedures in orthopedic surgery, with its utilization rising by approximately 55% over the past decade [103]. However, the COVID-19 pandemic exposed systemic inefficiencies across various economic sectors, compelling many to adapt to abrupt digital transformation. In response, alongside institutional efforts, numerous organizations worldwide capitalized on this opportunity to modernize. They implemented a range of e-health solutions, promoted telemedicine adoption, and piloted diverse telemedicine services [36].

Some authors hypothesize that VR may serve as a valuable adjunct in the management of postoperative pain [104]. VR enables users to experience an immersive environment that alters their immediate surroundings while isolating them from the external environment. It fully engages the patient’s auditory, visual, and proprioceptive sensory systems. One theory suggests that complete immersion in a VR setting may limit the brain’s capacity to process nociceptive signals, thereby reducing the perception of pain in the postoperative period, especially through gamification [105]. The concept of gamification is grounded in the application of “game design elements within non-gaming contexts” as a strategy to enhance motivation and encourage participation [106]. Positive effects have been documented across various domains of disability, including idiopathic scoliosis and stroke rehabilitation. However, this concept has been introduced relatively recently, and only a limited number of studies have explored it to date [107].

Despite significant advancements in orthopedic rehabilitation, several critical gaps in research remain unaddressed, posing challenges to the optimization and generalization of therapeutic interventions. One prominent limitation is the scarcity of long-term studies. While short- to medium-term outcomes are frequently reported, there is a lack of robust evidence regarding the durability of rehabilitation interventions over extended periods [108]. Understanding the long-term efficacy and sustainability of these approaches is crucial to ensuring that they provide enduring benefits and prevent future complications, such as recurrent injuries or degenerative changes.

Another major area requiring attention is the limited exploration of cost–benefit analyses in the implementation of advanced rehabilitation technologies. The adoption of tools such as digital medical devices and AI-driven solutions is growing; however, comprehensive evaluations of their economic feasibility and cost-effectiveness remain sparse. Such analyses are essential to determine whether these innovations justify their financial investment and to guide healthcare systems in resource allocation, particularly in low-resource settings [109].

Moreover, the validation of AI-based models in orthopedic rehabilitation presents a significant challenge. Many studies focus on homogeneous populations, often excluding diverse demographic and clinical groups. This lack of representation limits the generalizability of findings and raises concerns about the applicability of AI-driven interventions in real-world clinical practice. For instance, models trained on data from specific populations may fail to account for variability in age, gender, ethnicity, or comorbid conditions, leading to suboptimal outcomes when applied to broader patient cohorts [38].

## 7. Ethical Considerations and Critical Aspects

In medicine, AI is reshaping the landscape of diagnostics, surgical precision, and personalized care. Despite its transformative potential, AI in orthopedics raises significant ethical concerns that warrant careful examination and regulatory oversight.

One primary ethical consideration is data privacy. AI systems in orthopedics rely on extensive datasets, including electronic health records (EHRs), imaging studies, and wearable device outputs. These systems require strict safeguards to protect sensitive patient information from unauthorized access and misuse. Instances such as the 2015 DeepMind and UK National Health Service agreement—later criticized for breaching data protection laws—highlight the need for robust privacy protocols. Compliance with legal frameworks like GDPR and HIPAA is essential to maintaining patient trust and ensuring ethical data handling [110].

Algorithmic bias presents another challenge. AI models often inherit biases from the data used to train them. In orthopedics, this could result in unequal diagnostic or treatment outcomes for certain patient demographics. For example, algorithms trained predominantly on data from a single ethnic or gender group may fail to generalize effectively, disadvantaging underrepresented populations. Addressing this issue requires diversified training datasets and regular performance audits to identify and mitigate bias [111].

Cybersecurity vulnerabilities pose risks not only to patient safety but also to the integrity of medical decision-making. AI systems, particularly those integrated into surgical robotics or diagnostic platforms, are susceptible to adversarial attacks. Such breaches could alter diagnostic outputs or disrupt surgical planning, leading to adverse patient outcomes. Ensuring the security of AI infrastructures through encryption, multi-factor authentication, and continuous system monitoring is critical to countering these threats.

Another pressing concern is patient acceptance of AI technologies. While clinicians value AI for its precision and efficiency, patients may perceive it as impersonal or unreliable. The potential reduction in human interaction during medical consultations could undermine patient trust. Clear communication about AI’s role—as an assistive rather than autonomous tool—is vital to fostering acceptance [112]. Furthermore, involving patients in decision-making processes and providing opportunities for informed consent can enhance their confidence in AI-driven interventions.

The impact of AI on clinical skills must also be addressed. Automation of diagnostic and surgical tasks could lead to a decline in practitioner expertise over time, a phenomenon known as “deskilling” [113]. Ensuring that clinicians retain hands-on experience and decision-making abilities is essential to maintaining quality care, particularly in scenarios where AI tools fail or are unavailable [114].

Finally, the concept of accountability in AI-driven healthcare requires careful consideration. Determining liability in cases of errors—whether attributed to clinicians, developers, or institutions—is a complex issue. Clear guidelines must delineate responsibility for AI outcomes, balancing innovation with accountability [115].

While fully autonomous medical practice remains a distant prospect, AI in orthopedics is advancing rapidly within the framework of augmented intelligence. Human oversight remains critical for ensuring ethical use, interpreting AI-generated insights, and addressing system limitations [101]. As the field progresses, ongoing debates and refinements in regulatory standards will be essential to navigating emerging ethical dilemmas and securing AI’s place as a transformative yet responsible force in orthopedic care.

## 8. Current Barriers

Barriers to the widespread adoption of AI and robotics in THA include several critical challenges. One significant hurdle is cost-effectiveness, as the initial investment in advanced technologies, such as robotic systems and AI-powered tools, can be prohibitively high for many healthcare institutions. This economic barrier is compounded by the need for ongoing maintenance, software updates, and associated costs. Additionally, the implementation of these technologies requires extensive training for surgeons and operating room staff to ensure proficiency, which can further strain resources and extend the time required for integration into routine practice. Regulatory challenges also play a vital role, as obtaining approval for new technologies involves navigating complex and often lengthy processes that vary by region. These regulatory barriers can delay the availability of innovative tools and hinder their adoption. Addressing these issues is essential to realizing the full potential of AI and robotics in improving outcomes and efficiency in THA [116,117].

Robotic systems indeed require substantial upfront investment, with costs including installation, maintenance, and preoperative imaging, such as CT scans. These scans expose patients to higher radiation levels; however, advancements in scanning techniques, such as those highlighted by Tarwala et al., have shown promise in reducing radiation doses without compromising image quality [16]. Furthermore, the financial burden of robotic systems, with initial setup costs often exceeding USD one million, may seem daunting. Yet, competition in the market is expected to lower these costs. While these expenses can be significant, long-term savings may offset them, as robotic systems are associated with reduced complication and reoperation rates, lower rehabilitation needs, and shorter hospital stays. For instance, Pierce et al. demonstrated a cost-saving of approximately USD 785 for robotic THA when compared to conventional approaches [6]. Similarly, Maldonado et al. found robotic THA to be cost-effective over five years, saving up to USD 1810 for privately insured patients [20]. Exploring these potential cost offsets and the financial implications of reduced hospital resource consumption could enhance the discussion on accessibility.

Overcoming cost and technological limitations in robotic THA necessitates a multi-faceted strategy. First, fostering competition among manufacturers may reduce the initial capital required for robotic systems. Additionally, focusing on long-term cost efficiencies, such as fewer complications, shorter hospital stays, and reduced postoperative care requirements, may help justify the investment. Policies encouraging wider adoption, such as subsidies or incentives for hospitals implementing robotic technologies, could also play a role. Training surgeons and staff efficiently and incorporating advanced imaging technologies with reduced radiation exposure are key steps in addressing these limitations [118].

## 9. Conclusions

Advanced technologies such as AI, robotics, VR, and AR are transforming THA by enhancing diagnosis, surgical precision, and rehabilitation. AI improves preoperative planning, implant identification, and risk assessment, while robotic-assisted surgery ensures accurate implant placement and reduced complications. VR and AR enhance surgical training, intraoperative navigation, and patient engagement in rehabilitation, offering more accessible and efficient care.

Despite challenges like cost and limited adoption among older populations, these innovations hold immense potential to improve outcomes and streamline orthopedic workflows. Continued research, refinement, and integration into clinical practice are essential to fully harness their benefits. As these technologies evolve, they are set to redefine standards in orthopedic surgery, delivering more personalized and effective care.

Future studies should focus on conducting large-scale, multicenter trials to validate the long-term efficacy and safety of these technologies in diverse clinical settings. Research efforts should also prioritize the development of cost-effective solutions and simplified interfaces tailored to elderly patients, addressing the digital divide and ensuring equitable access. Furthermore, integrating multimodal data, including imaging, genetic, and clinical information, into AI models could enhance predictive capabilities and enable even more personalized care. Lastly, studies exploring the ethical implications of AI and robotics in orthopedics, such as data privacy and algorithmic bias, will be essential in fostering patient trust and ensuring responsible adoption of these transformative tools. By addressing these gaps, future research can pave the way for a new era of precision medicine in orthopedic surgery.

**Figure 1:**
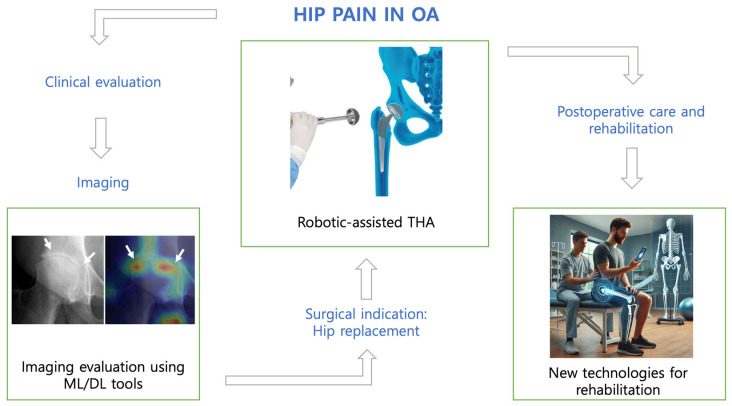
Clinical application workflow of key artificial intelligence tools and new technologies [OA: osteoarthritis; ML: machine learning; DL: deep learning; THA: total hip arthroplasty].

Figure 1 illustrates a clinical application workflow of key artificial intelligence tools and new technologies, spanning from preoperative care to rehabilitation, with the potential for further advancements in the future.

## Figures and Tables

**Table 1:** Comprehensive overview of AI applications in total hip arthroplasty [THA: total hip arthroplasty; AI: artificial intelligence; OA: osteoarthritis; DRR: digitally reconstructed radiograph; CNN: convolutional neural network; VR: virtual reality; AR: augmented reality; DL: deep learning; ML: machine learning].

Application Area	Specific AI Use	Impact	Key Examples
Diagnosis	Automated grading of hip OA severity	Improved diagnostic accuracy and reliability	DL models (e.g., DRR-based systems) [9,10,11]
Preoperative Planning	Predicting hip joint center; implant positioning	Reduced variability in planning; enhanced precision	AI models predicting hip joint center, 3D imaging [12,13,14,15]
Surgical Assistance	Real-time intraoperative feedback during robotic-assisted THA	Enhanced implant placement precision; reduced complications	Robotic systems (image-based vs. imageless) [16,17,18,19,20,21]
Risk Stratification	Predicting postoperative complications based on patient-specific data	Personalized risk assessment; improved decision-making	ML algorithms [22,23]
Implant Identification	Automated recognition of implant designs from imaging	Reduced planning time; streamlined revision	CNN-based implant recognition tools [24,25,26,27,28,29]
Rehabilitation	VR and AR for guided exercises and patient engagement	Improved adherence to recovery programs; enhanced patient satisfaction	AR-guided systems (e.g., HoloLens) and VR platforms [30,31,32,33,34,35,36]
Outcome Prediction	Forecasting long-term implant success and risk of revision	Proactive patient management; improved long-term outcomes	AI models analyzing radiological and clinical data [37,38]

## Data Availability

No new data were created or analyzed in this study.
